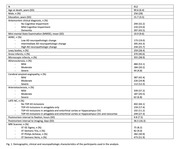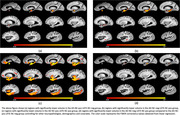# Investigating Spatial Characteristics of Brain Atrophy in Alzheimer's and LATE neuropathology

**DOI:** 10.1002/alz.093770

**Published:** 2025-01-09

**Authors:** Khalid Saifullah, Abdur Raquib Ridwan, David A. Bennett, Julie A. Schneider, Konstantinos Arfanakis

**Affiliations:** ^1^ Illinois Institute of Technology, Chicago, IL USA; ^2^ Rush Alzheimer's Disease Center, Chicago, IL USA; ^3^ Rush Alzheimer's Disease Center, Rush University Medical Center, Chicago, IL USA

## Abstract

**Background:**

Alzheimer’s disease neuropathologic change (AD‐NC) and limbic‐predominant age‐related TDP‐43 encephalopathy neuropathologic change (LATE‐NC) are common in older adults and have been associated with brain atrophy, cognitive decline, and dementia. Since AD‐NC and LATE‐NC are often comorbid and due to the fact that LATE‐NC can only be detected at autopsy, in this work, we combined deformation‐based morphometry (DBM) on ex‐vivo brain MRI and detailed neuropathological evaluation in a large number of community‐based older adults to investigate the difference in brain atrophy patterns associated with AD‐NC and LATE‐NC.

**Method:**

Cerebral hemispheres from 912 older adults participating in four longitudinal, clinical‐pathologic cohort studies of aging were included in this work: MAP, ROS, MARS, and AA Core of the Rush Alzheimer’s Disease Research Center (Rush ADRC) (Figure 1). All hemispheres were imaged ex‐vivo on 3T clinical MRI scanners approximately 1‐month postmortem while immersed in 4% formaldehyde solution. T2‐weighted images of all hemispheres were non‐linearly registered and logarithm of the Jacobian determinant (LogJ) of the deformation field was calculated in each voxel. Following ex‐vivo MRI, the pathologies that were assessed were AD‐NC, LATE‐NC, Lewy bodies, gross infarcts, microscopic infarcts, arteriolosclerosis, atherosclerosis, and cerebral amyloid angiopathy. AD‐NC‐pos was defined as moderate or severe AD‐NC according to the NIA‐AA criteria, and LATE‐NC‐pos was defined as LATE‐NC stages 2 or 3. Voxel‐wise linear regression was used to test the association of the deformations observed in the smoothed log Jacobian maps with the four different groups, controlling for all other neuropathologies, demographics, postmortem intervals, and scanner. We used 5000 permutations, and statistical significance was set at p<0.05 after family wise error (FWE) correction.

**Result:**

Both the AD‐NC‐pos LATE‐NC‐neg group (Figure 2a) as well as the AD‐NC‐neg LATE‐NC‐pos group (Figure 2b) were associated with lower tissue volume mainly in medial temporal lobe structures. The AD‐NC‐pos LATE‐NC‐pos group (Figure 2c) showed substantially lower volume in the temporal, frontal, and parietal lobes. Interestingly, the AD‐NC‐neg LATE‐NC‐pos group showed lower volume in the anterior portion of the hippocampus than the AD‐NC‐pos LATE‐NC‐neg group (Figure 2d).

**Conclusion:**

The findings suggest that in the presence of LATE‐NC, the volume of the hippocampus cannot serve as a marker of AD‐NC.